# Supplementary material for: Weather Patterns Associated with DON Levels in Norwegian Spring Oat Grain: A Functional Data Approach
Source: Plants (Basel). 2021 Dec 27;11(1):73. doi: 10.3390/plants11010073 (PMC8747184; doi:10.3390/plants11010073)
Supplement: Supplementary file 1 [file plants-11-00073-s001.zip › plants-1492774-supplementary.pdf]

Weather patterns associated with DON levels in Norwegian spring oat grain: a functional data approach

Anne-Grete Roer Hjelkrem[1]*, Heidi Udnes Aamot[2], Morten Lillemo[3], Espen Sannes Sørensen[4], Guro Brodal[2], Aina Lundon Russenes[1], Simon G. Edwards[5] and Ingerd Skow Hofgaard[2]

[1] Division of Food Production and Society, Norwegian Institute of Bioeconomy Research (NIBIO), 1431 Ås, Norway
[2] Division of Biotechnology and Plant Health, Norwegian Institute of Bioeconomy Research (NIBIO), 1431 Ås, Norway
[3] Department of Plant Sciences, Norwegian University of Life Sciences (NMBU), 1432 Ås, Norway
[4] Graminor Breeding, 2322 Ridabu, Norway
[5] Centre for Integrated Pest Management, Harper Adams University, Newport, Shropshire TF 10 8NB, UK
* Correspondence: anne-grete.hjelkrem@nibio.no

Supplementary Materials:

3 Tables (S1-3) and 12 Figures (S1-11).

Table S1: Specifications of the different Gompertz models[1] evaluated, with definition of the adjustemt of the degree day accumulation, the number and specification of the fixed values and of the free unknown model parameters.

Model	Degree day adjustment	Environmental variables[5]	Fixed values	Unknown parameters
**Model 1**	$\sum_{\boldsymbol{i}}(\boldsymbol{T_i}-\boldsymbol{T_b})_+$	1 (T)	GS_{max} = 99, T_b = 0 °C	2 (α, β)
**Model 2**	$\sum_{\boldsymbol{i}}(\boldsymbol{T_i}-\boldsymbol{T_b})_+$	1 (T)	T_b = 0 °C	3 (GS_{max}, α, β)
**Model 3**	$\sum_{\boldsymbol{i}}(\boldsymbol{T_i}-\boldsymbol{T_b})_+$	1 (T)	T_b = 5 °C	3 (GS_{max}, α, β)
**Model 4**	$\sum_{\boldsymbol{i}}(\boldsymbol{T_i}-\boldsymbol{T_b})_+$	1 (T)		4 (GS_{max}, α, β, T_b)
**Model 5**	$\sum_{i\in A}(T_i-\boldsymbol{T_{b1}})_+ + \sum_{i\in B}(T_i-\boldsymbol{T_{b2}})_+$[2]	1 (T)	T_{b1} = 0 °C, T_{b2} = 9 °C, GS_{thres} = 55	3 (GS_{max}, α, β)
**Model 6**	$\sum_{i\in A}(T_i-\boldsymbol{T_{b1}})_+ + \sum_{i\in B}(T_i-\boldsymbol{T_{b2}})_+$[2]	1 (T)	GS_{thres} = 55	5 (GS_{max}, α, β, T_{b1}, T_{b2})
**Model 7**	$\sum_{i\in A}(T_i-\boldsymbol{T_{b1}})_+ + \sum_{i\in B}(T_i-\boldsymbol{T_{b2}})_+$[2]	1 (T)	GS_{thres} = 65	5 (GS_{max}, α, β, T_{b1}, T_{b2})
**Model 8**	$\sum_i sine(T_{max}, T_{min}, T_b)_+$[3]	2 (T_{min}, T_{max})	T_b = 0 °C	3 (GS_{max}, α, β)
**Model 9**	$\sum_{\boldsymbol{i}}\left(\frac{T_{upper}-T_i}{T_{upper}-T_{opt}}\right)\cdot\left(\frac{T_i-T_b}{T_{opt}-T_b}\right)^{\left(\frac{T_{opt}-T_{min}}{T_{upper}-T_{min}}\right)}$[4] [4]	1 (T)		6 (GS_{max}, α, β, T_b, T_{upper}, T_{opt})
**Model 10**	$\sum_i \lambda_i/24\cdot(T_i-\boldsymbol{T_b})_+$	2 (T, λ)	T_b = 0 °C	3 (GS_{max}, α, β)
**Model 11**	$\sum_i \lambda_i/24\cdot(T_i-\boldsymbol{T_b})_+$	2 (T, λ)	T_b = 5 °C	3 (GS_{max}, α, β)
**Model 12**	$\sum_i Ph_i\cdot(T_i-\boldsymbol{T_b})_+$	2 (T, Ph)	T_b = 0 °C	3 (GS_{max}, α, β)
**Model 13**	$\sum_{i\in A}\lambda_i/24\cdot(T_i-\boldsymbol{T_b})_+ + \sum_{i\in B}(T_i-\boldsymbol{T_b})_+$[2]	2 (T, λ)	T_b = 0 °C, GS_{thres} = 55	3 (GS_{max}, α, β)
**Model 14**	$\sum_{i\in A}\lambda_i/24\cdot(T_i-\boldsymbol{T_b})_+ + \sum_{i\in B}(T_i-\boldsymbol{T_b})_+$[2]	2 (T, λ)	T_b = 0 °C, GS_{thres} = 65	3 (GS_{max}, α, β)
**Model 15**	$\sum_{i\in A}\lambda_i/24\cdot(T_i-\boldsymbol{T_b})_+ + \sum_{i\in B}(T_i-\boldsymbol{T_b})_+$[2]	2 (T, λ)	T_b = 0 °C	4 (GS_{max}, α, β, GS_{thres})
**Model 16**	$\sum_{i\in A}\lambda_i/24\cdot(T_i-\boldsymbol{T_b})_+ + \sum_{i\in B}(T_i-\boldsymbol{T_b})_+$[2]	2 (T, λ)	T_b = 5 °C, GS_{thres} = 65	3 (GS_{max}, α, β)
**Model 17**	$\sum_{i\in A}Ph_i\cdot(T_i-\boldsymbol{T_b})_+ + \sum_{i\in B}(T_i-\boldsymbol{T_b})_+$[2]	2 (T, Ph)	T_b = 0 °C, GS_{thres} = 55	3 (GS_{max}, α, β)
**Model 18**	$\sum_{i\in A}Ph_i\cdot(T_i-\boldsymbol{T_b})_+ + \sum_{i\in B}(T_i-\boldsymbol{T_b})_+$[2]	2 (T, Ph)	T_b = 0 °C	4 (GS_{max}, α, β, GS_{thres})

The abbreviations of the weather factors and the model parameters are described in the Materials and methods section

[1] $GS_i = GS_{max}\cdot e^{-\alpha\cdot e^{-\beta\cdot DD_i}}$, *where GS_i is the estimated growth stage at day I, GS_{max} is maximum growth stage to reach, α sets the displacement along the x-axis, β sets the growth rate and DD_i is the adjusted degree-day accumulation from sowing to day i.*

[2] *A is the subset of days before a threshold phenologial stage (GS_{thres}), B is the subset of days thereafter*

[3] *Baskerville and Emin (1968)*

[4] *Yan and Hunt (1999)*

[5] *T – mean daily air temperature, T_{min} – minimum daily air temperature, T_{max} – maximum daily air temperature, λ – photoperiod, Ph – photoperiod in accordance with Olesen et al. (2012)*

Table S2: Estimated root mean square error of validation (RMSEV) for the Belinda variety, given for each proposed model as mean value over the splits and for each split in the cross site-year validation separately.

	Apelsvoll 2015	Apelsvoll 2016	Apelsvoll 2017	Ås 2018	Apelsvoll 2018	Staur 2018	Bjørke 2018	Ås 2019	Apelsvoll 2019	Bjørke 2019	Staur 2019	Mean
**Model 1**	2.98	4.35	2.79 [1]	3.81	5.17	3.64	4.86	4.39	5.87 [2]	2.93	5.08	4.17
**Model 2**	2.93	4.29	2.75 [1]	3.78	5.14	3.71	4.90	4.46	5.88 [2]	3.00	5.11	4.18
**Model 3**	10.80 [2]	5.94	2.86	3.28	4.89	7.38	7.11	4.48	4.38	2.37 [1]	3.92	5.22
**Model 4**	4.37	4.59	2.67 [1]	3.27	4.86	4.69	4.95	4.32	5.61 [2]	2.91	4.88	4.28
**Model 5**	4.25	5.49	4.51 [1]	4.77	3.92	5.33	6.17	6.32	7.00 [2]	5.47	6.36	5.43
**Model 6**	4.13	4.52	2.62 [1]	3.19	4.89	4.82	4.90	4.34	5.65 [2]	3.04	4.86	4.25
**Model 7**	4.28	4.44	2.51 [1]	3.06	4.87	4.59	4.89	4.40	5.80 [2]	2.99	5.08	4.26
**Model 8**	3.52	3.73	2.95 [1]	5.08	5.31	2.97	5.26	4.49	6.60 [2]	3.21	5.17	4.39
**Model 9**	7.00	4.14	3.15 [1]	3.47	5.04	4.64	5.30	4.71	5.67 [2]	3.44	4.35	4.63
**Model 10**	2.73	3.96	2.69 [1]	3.88	5.07	3.67	4.81	5.36	5.98 [2]	3.35	5.28	4.25
**Model 11**	10.80 [2]	5.48	2.70 [1]	3.03	4.35	7.29	7.48	5.16	4.53	2.72	4.32	5.26
**Model 12**	2.67	3.68	2.76 [1]	3.92	5.02	3.81	4.88	6.10	6.14 [2]	3.71	5.54	4.39
**Model 13**	3.20	4.33	2.86 [1]	3.93	5.61	3.62	4.87	4.75	5.88 [2]	3.21	4.71	4.27
**Model 14**	3.17	4.32	2.88 [1]	4.11	5.35	3.63	5.04	5.00	5.99 [2]	3.29	4.88	4.33
**Model 15**	2.95	4.35	2.95	3.82	5.78	3.18	4.58	4.54	4.94	2.05 [1]	9.46 [2]	4.26
**Model 16**	10.89	5.49	2.83 [1]	3.26	4.59	7.01	7.61	4.77	5.93	4.47	14.85 [2]	6.47
**Model 17**	3.11	4.15	2.89 [1]	4.15	5.70	3.43	4.96	4.95	5.87 [2]	3.58	3.87	4.24
**Model 18**	2.97	4.32	2.89 [1]	3.70	5.90	3.12	4.84	4.40	5.75 [2]	3.32	3.98	4.11

[1]The lowest RMSEV over the splits

[2]The largest RMSEV over the splits

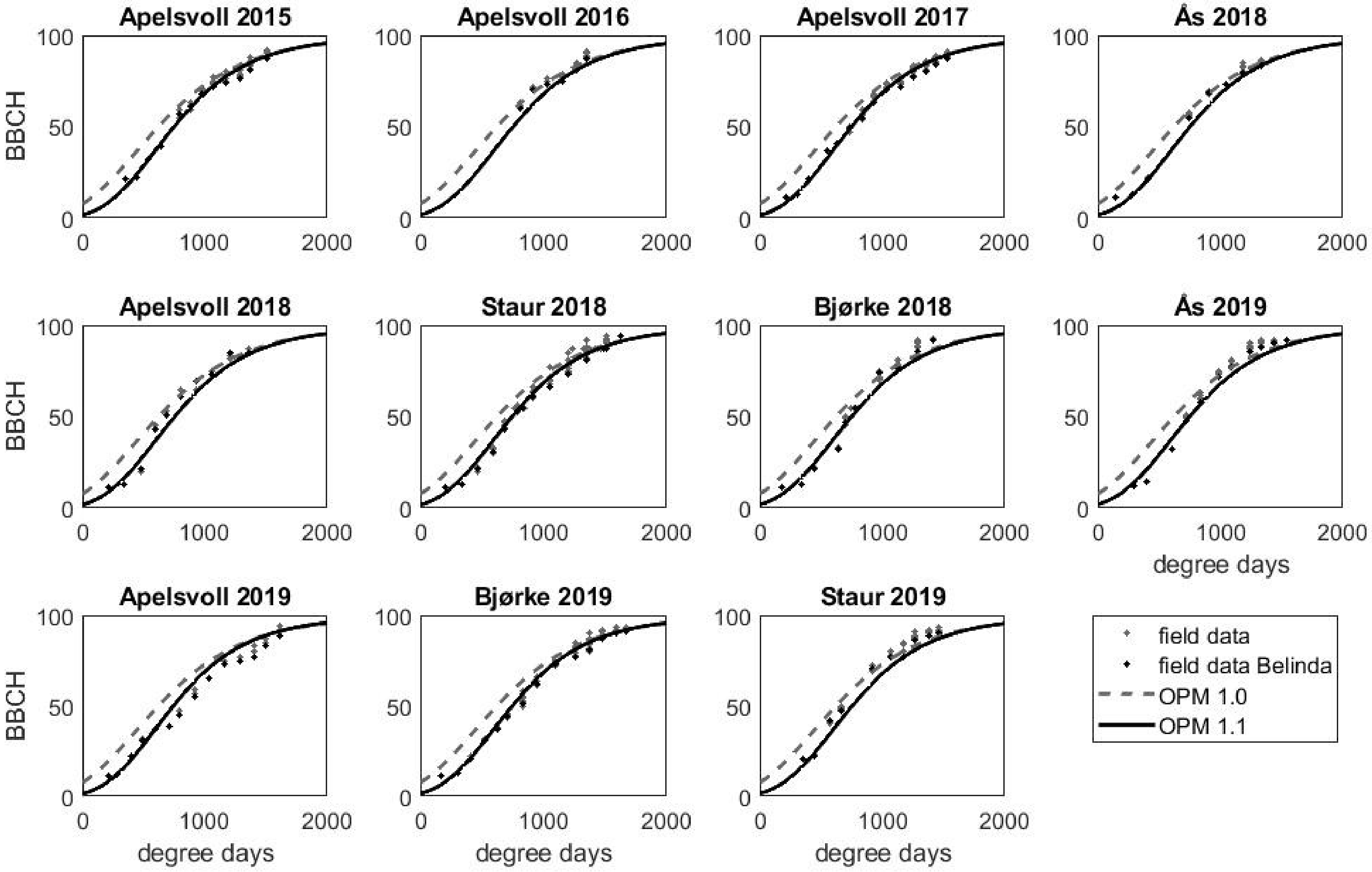

Figure S1: The previous OPM 1.0 model together with the new OPM 1.1 model, plotted together with the validation data for each split in the cross site-year validation separately.

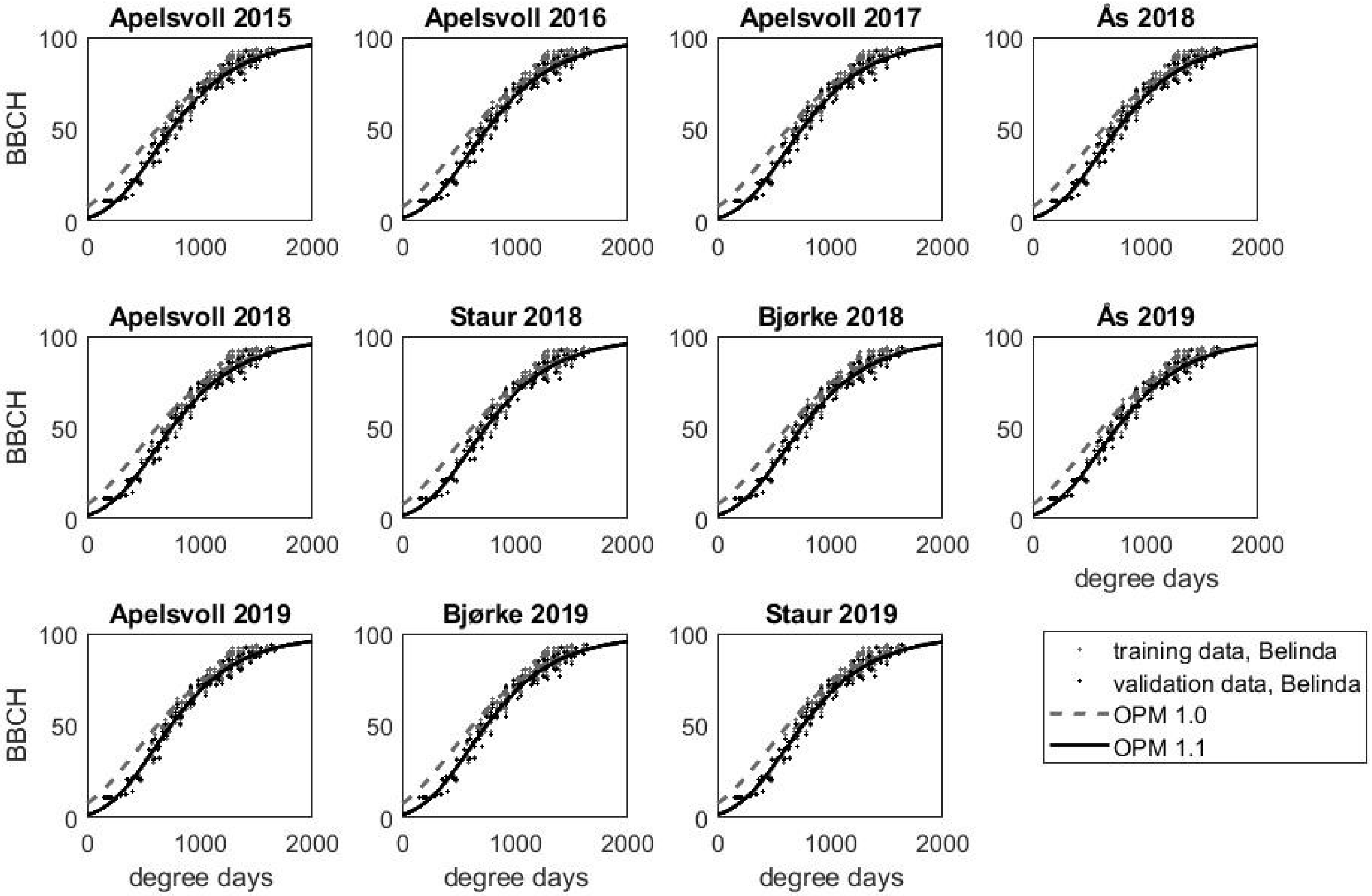

Figure S2: The previous OPM 1.0 model together with the new OPM 1.1 model, plotted together with the training and the validation data of the Belinda variety, for each split in the cross site-year validation separately.

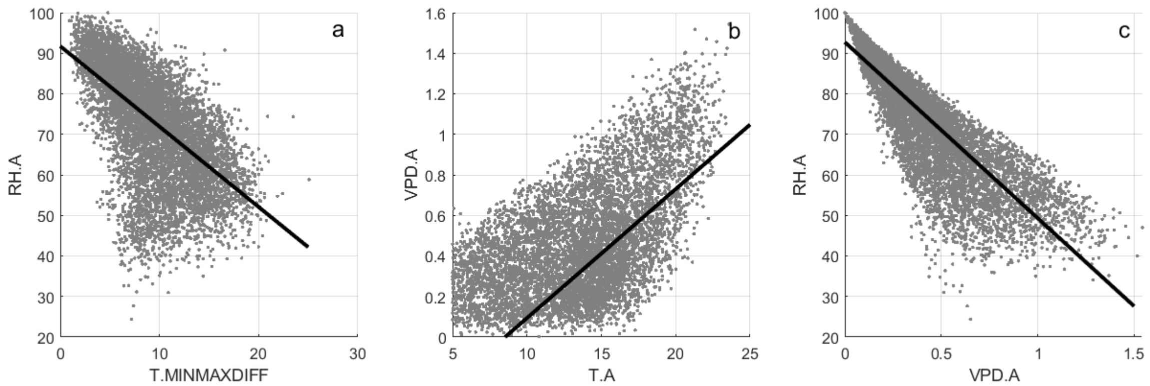

Figure S3: a) Average daily relative humidity (RH.A) given as a function of daily difference between maximum and minimum hourly observed air temperature (T.MINMAXDIFF), b) average daily vapor pressure deficit (vpd.A) given as a function of average daily temperature (T.A) and, c) RH.A given as a function of vpd.A using daily observations during a period from 90 days prior to 30 days posterior to estimated flowering of oats

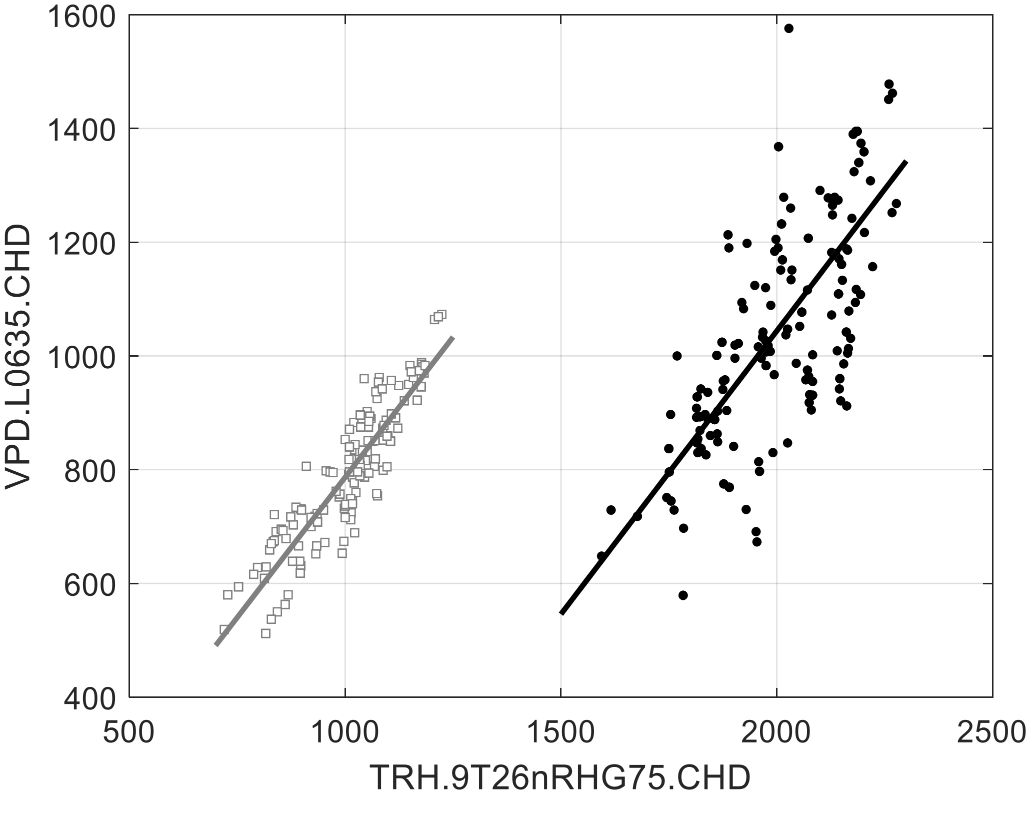

Figure S4: Accumulated number of hours with vapor pressure deficit lower than 0.635 kPa (VPD.L0635.CHD) as a function of accumulated number of hours with temperature between 9 and 26 °C combined with relative humidity greater than 75% (TRH.9T26nRHL75.CHD) during a period from 90 days prior to 30 days posterior to estimated flowering (black circles) and during a period from 30 days prior to 30 days posterior to estimated flowering (gray squares).

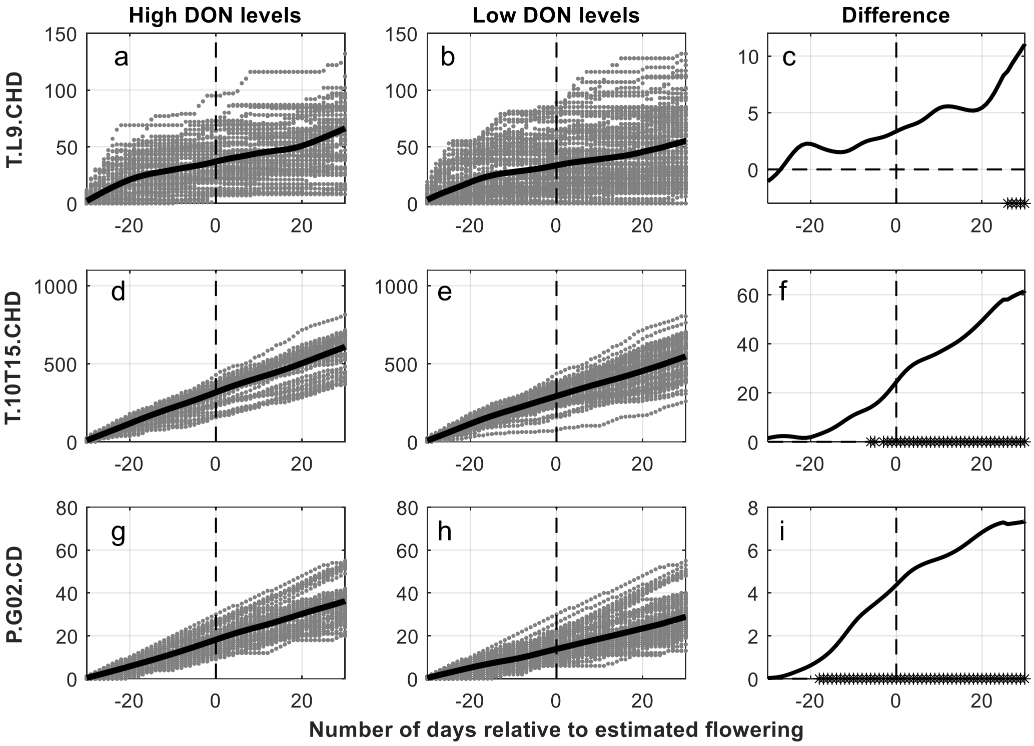

Figure S5: Calculated output data (gray) for five weather variables versus the number of days relative to estimated flowering in oats, grouped according to levels of deoxynivalenol (High DON levels, first column of panels: DON ≥ 500 µg pr kg harvested oat. Low DON levels, second column of panels: DON < 500 µg per kg harvested oat). Black lines indicate the functional mean values of the smooth functions derived from individual sets of weather data. In the third column of panels, the difference between the functional mean curves (high - low) are given as black lines with asterisk at the bottom of each panel indicating significant differences between the mean values (5% level). Weather data is presented from 30 days prior to 30 days posterior to flowering (dashed line at 0), estimated by the use of phenological model (OPM1.1). In all panels, the two dash-dotted lines indicate the start and end of the period of sowing for the different fields. T.L9.CHD = cumulative number of hours with temperature lower than 9 °C, T.20T15.CHD = cumulative number of hours with temperature between 10 and 15 °C and P.G02.CD = cumulative days with precipitation greater than 0.2 mm.

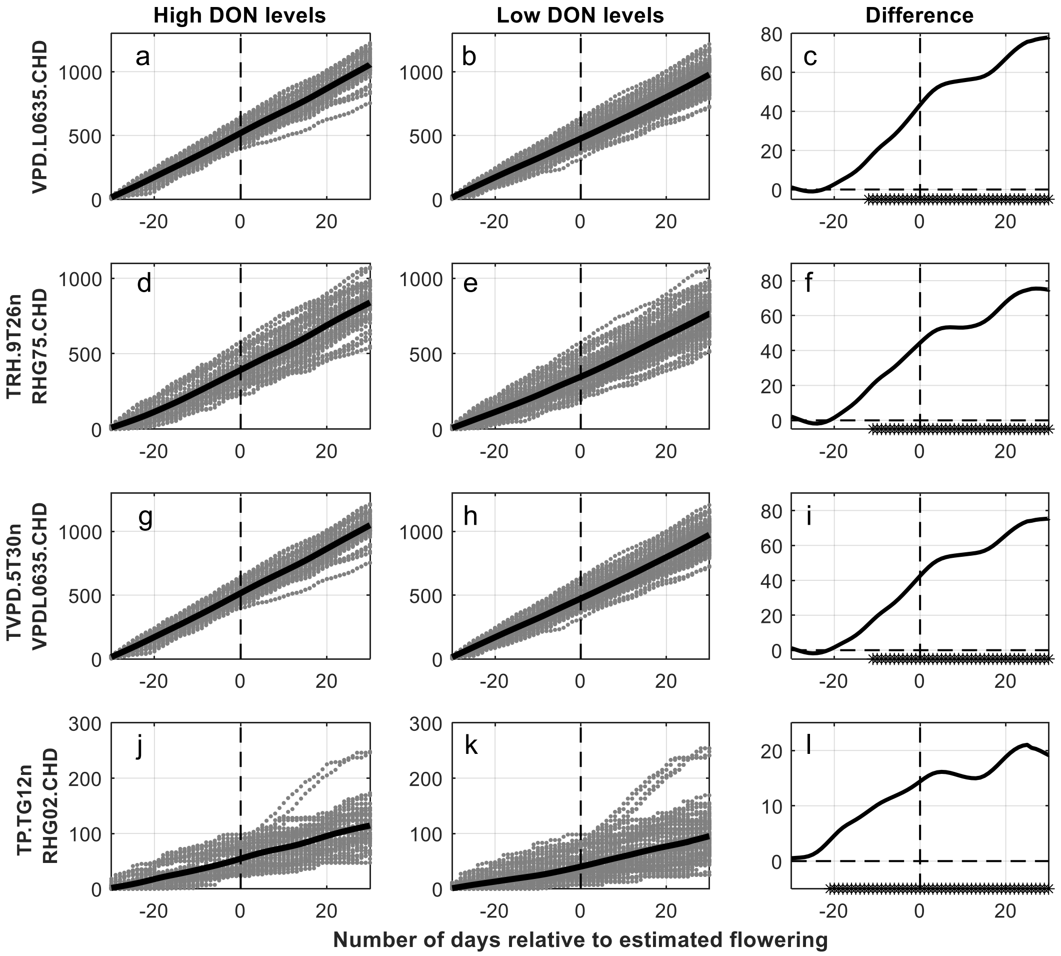

Figure S6: Calculated output data (gray) for five weather variables versus the number of days relative to estimated flowering in oats, grouped according to levels of deoxynivalenol (High DON levels, first column of panels: DON ≥ 500 µg pr kg harvested oat. Low DON levels, second column of panels: DON < 500 µg per kg harvested oat). Black lines indicate the functional mean values of the smooth functions derived from individual sets of weather data. In the third column of panels, the difference between the functional mean curves (high - low) are given as black lines with asterisk at the bottom of each panel indicating significant differences between the mean values (5% level). Weather data is presented from 30 days prior to 30 days posterior to flowering (dashed line at 0) estimated by the use of phenological model (OPM1.1). In all panels, the two dash-dotted lines indicate the start and end of the period of sowing for the different fields.TRH.9T26nRHG75.CHD = cumulative number of hours with temperature between 9 and 26 °C combined with a relative humidity greater than 75%, VPD.L0635.CHD = cumulative number of hours with vapour pressure deficit lower than 0.635 kPa, TVPD.5T30nVPDL0635.CHD = cumulative number of hours with temperature between 5 and 30 °C combined with vapour pressure deficit lower than 0.635 kPa and TP.TG12nPG02.CHD = cumulative number of hours with temperature above 12 °C combined with precipitation greater than 0.2 mm.

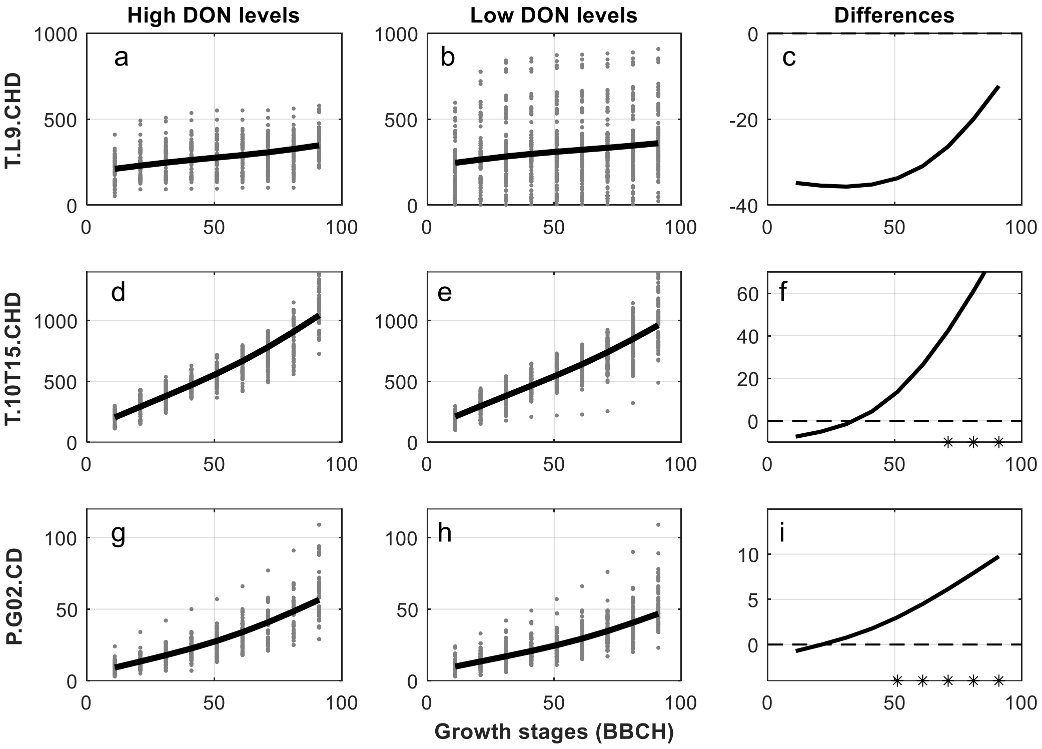

Figure S7: Calculated output data (gray) for five weather variables versus the estimated growth stages in oats, grouped according to levels of deoxynivalenol (High DON levels, first column of panels: DON ≥ 500 µg pr kg harvested oat. Low DON levels, second column of panels: DON < 500 µg per kg harvested oat). Black lines indicate the functional mean values of the smooth functions derived from individual sets of weather data. In the third column of panels, the difference between the functional mean curves (high - low) are given as black lines with asterisk at the bottom of each panel indicating significant differences between the mean values (5% level). Weather data is presented relative to the following estimated growth stages: BBCH 11, 21, 31, 41, 51, 61, 71, 81 and 91. T.L9.CHD = cumulative number of hours with temperature lower than 9 °C, T.20T15.CHD = cumulative number of hours with temperature between 10 and 15 °C and P.G02.CD = cumulative days with precipitation greater than 0.2 mm.

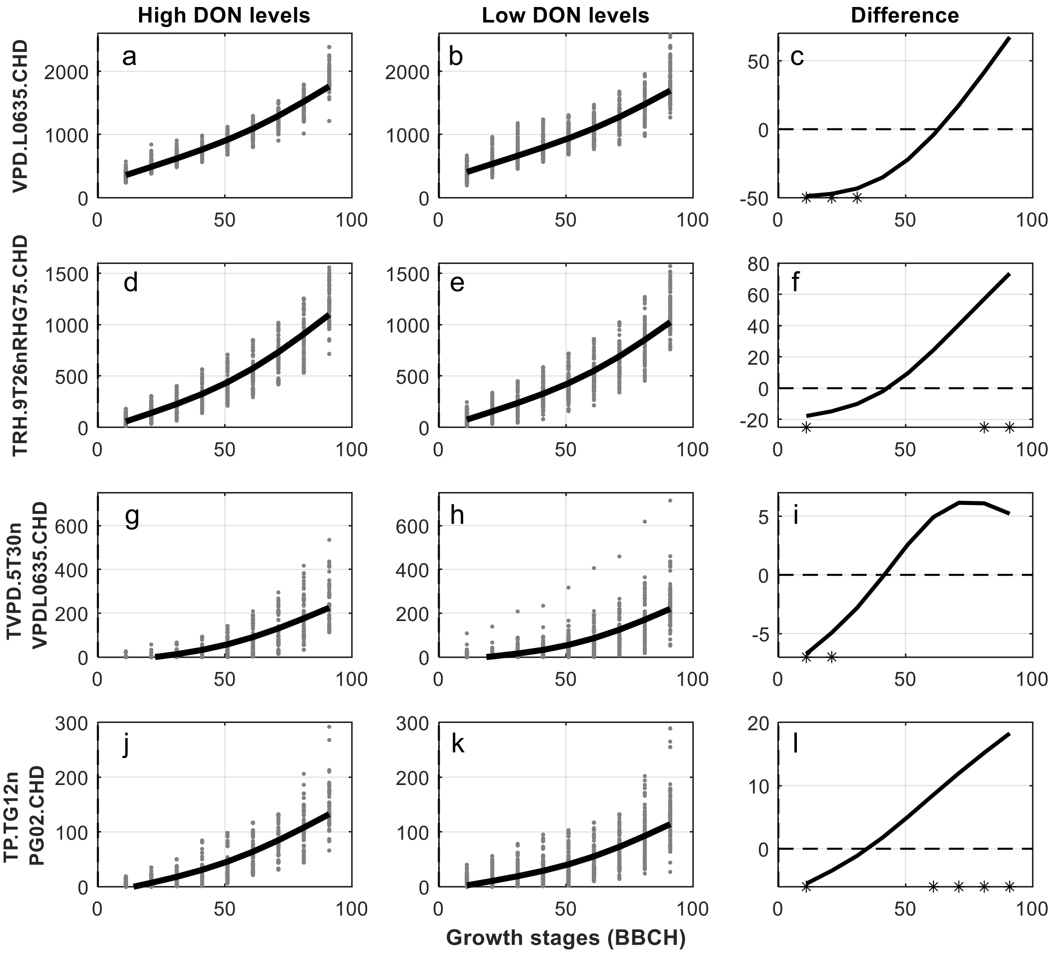

Figure S8: Calculated output data (gray) for five weather variables versus the estimated growth stages in oats, grouped according to levels of deoxynivalenol (High DON levels, first column of panels: DON ≥ 500 µg pr kg harvested oat. Low DON levels, second column of panels: DON < 500 µg per kg harvested oat). Black lines indicate the functional mean values of the smooth functions derived from individual sets of weather data. In the third column of panels, the difference between the functional mean curves (high - low) are given as black lines with asterisk at the bottom of each panel indicating significant differences between the mean values (5% level). Weather data is presented relative to the following estimated growth stages: BBCH 11, 21, 31, 41, 51, 61, 71, 81 and 91. VPD.L0635.CHD = cumulative number of hours with vapour pressure deficit lower than 0.635 kPa, TRH.9T26nRHG75.CHD = cumulative number of hours with temperature between 9 and 26 °C combined with a relative humidity greater than 75%, TVPD.5T30nVPDL0635.CHD = cumulative number of hours with temperature between 5 and 30 °C combined with vapour pressure deficit lower than 0.635 kPa and TP.TG12nPG02.CHD = cumulative number of hours with temperature above 12 °C combined with precipitation greater than 0.2 mm.

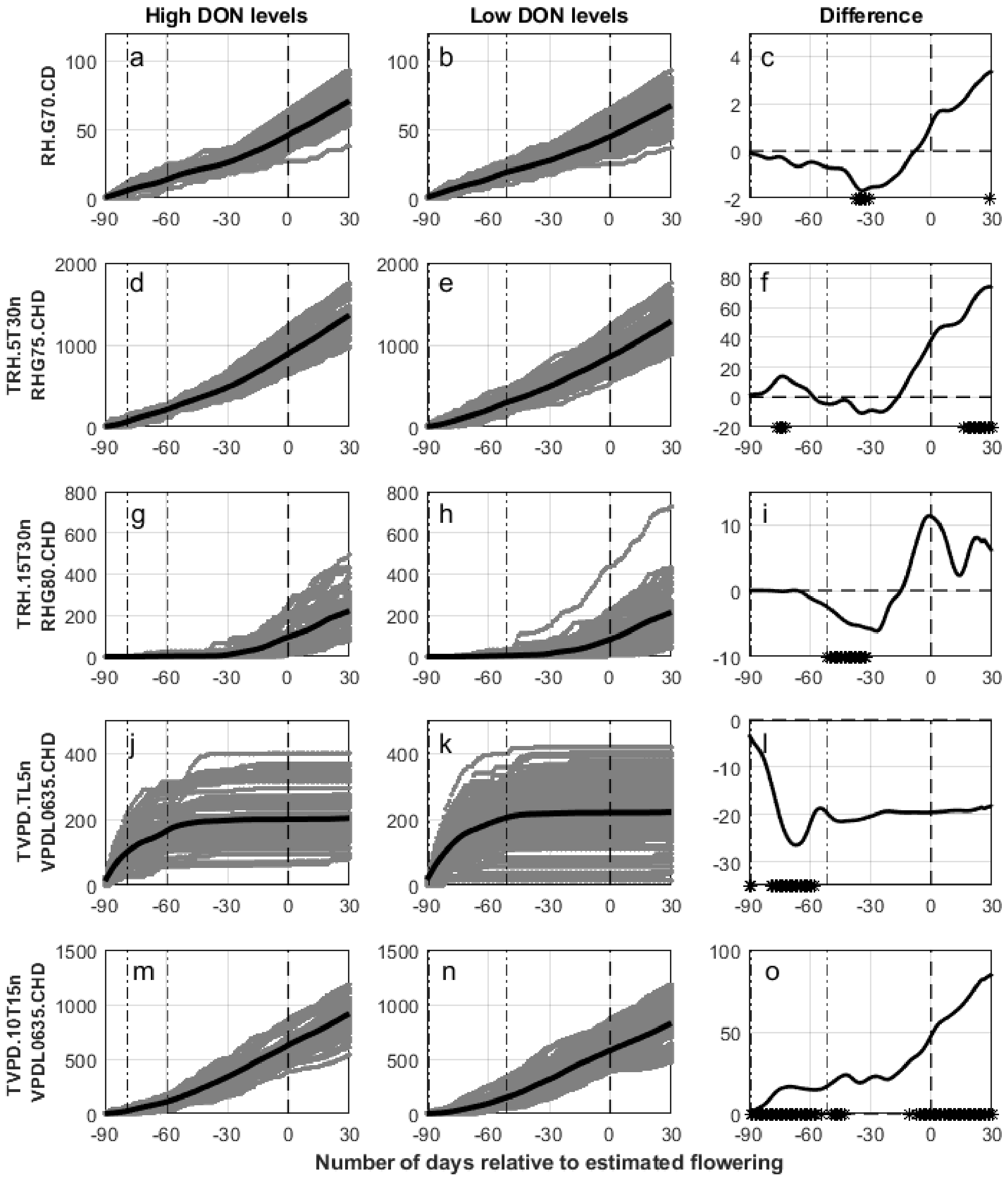

Figure S9: Calculated output data (gray) for five weather variables versus the number of days relative to estimated flowering in oats, grouped according to levels of deoxynivalenol (High DON levels, first column of panels: DON ≥ 500 µg pr kg harvested oat. Low DON levels, second column of panels: DON < 500 µg per kg harvested oat). Black lines indicate the functional mean values of the smooth functions derived from individual sets of weather data. In the third column of panels, the difference between the functional mean curves (high - low) are given as black lines with asterisk at the bottom of each panel indicating significant differences between the mean values (5% level). Weather data is presented from 90 days prior to 30 days posterior to flowering (dashed line at 0) estimated by the use of phenological model (OPM1.1). In all panels, the two dash-dotted lines indicate the start and end of the period of sowing for the different fields.RH.G70.CD = cumulative days with relative humidity greater than 70%, TRH.5T30nRHG75.CHD = cumulative number of hours with temperature between 5 and 30 °C combined with relative humidity greater than 75%, TRH.15T30nRHG80.CHD = cumulative number of hours with temperature between 15 and 30 °C combined with relative humidity greater than 80%, TVPD.TL5nVPDL0635.CHD = cumulative number of hours with temperature lower than 5 °C combined with vapor pressure deficit lower than 0.635 kPa and TVPD.10T15nVPDL0635.CHD = cumulative number of hours with temperature between 10 and 15 °C combined with vapor pressure deficit lower than 0.635 kPa.

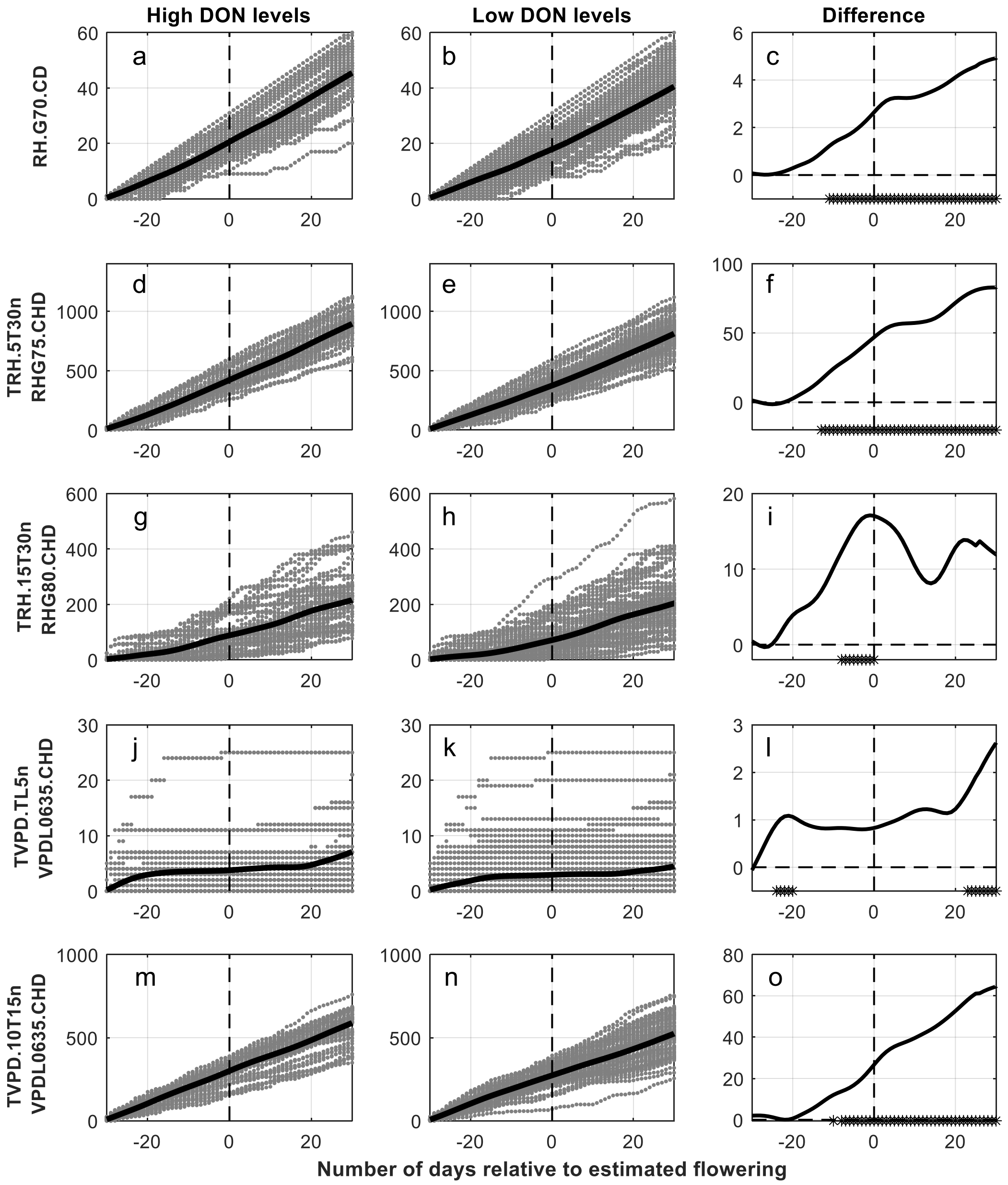

Figure S10: Calculated output data (gray) for five weather variables versus the number of days relative to estimated flowering in oats, grouped according to levels of deoxynivalenol (High DON levels, first column of panels: DON ≥ 500 µg pr kg harvested oat. Low DON levels, second column of panels: DON < 500 µg per kg harvested oat). Black lines indicate the functional mean values of the smooth functions derived from individual sets of weather data. In the third column of panels, the difference between the functional mean curves (high - low) are given as black lines with asterisk at the bottom of each panel indicating significant differences between the mean values (5% level). Weather data is presented from 30 days prior to 30 days posterior to flowering (dashed line at 0) estimated by the use of phenological model (OPM1.1). In all panels, the two dash-dotted lines indicate the start and end of the period of sowing for the different fields.RH.G70.CD = cumulative days with relative humidity greater than 70%, TRH.5T30nRHG75.CHD = cumulative number of hours with temperature between 5 and 30 °C combined with relative humidity greater than 75%, TRH.15T30nRHG80.CHD = cumulative number of hours with temperature between 15 and 30 °C combined with relative humidity greater than 80%, TVPD.TL5nVPDL0635.CHD = cumulative number of hours with temperature lower than 5 °C combined with vapor pressure deficit lower than 0.635 kPa and TVPD.10T15nVPDL0635.CHD = cumulative number of hours with temperature between 10 and 15 °C combined with vapor pressure deficit lower than 0.635 kPa.

Table S3: Overview of field observations of phenological oat growth stages.

**Site**	**Weather station**	**Year**	**Sowing date (day number)**	**BBCH period**	**Distance weather Station (km)**
Kapp	Apelsvoll	2015	24. April (114)	21-90	< 2
Kapp	Apelsvoll	2016	9. May (130)	60-90	< 2
Kapp	Apelsvoll	2017	10. May (130)	11-90	< 2
Ås	Ås	2018	22. May (142)	11-86	< 2
Kapp	Apelsvoll	2018	9. May (129)	11-87	< 2
Staur	Ilseng	2018	10. May (130)	11-94	10
Bjørke	Ilseng	2018	23. May (143)	11-92	< 1
Ås	Ås	2019	29. May (149)	12-92	< 2
Kapp	Apelsvoll	2019	4. May (124)	11-94	< 2
Bjørke	Ilseng	2019	17. May (137)	11-93	< 1
Staur	Ilseng	2019	4. June (155)	21-93	10

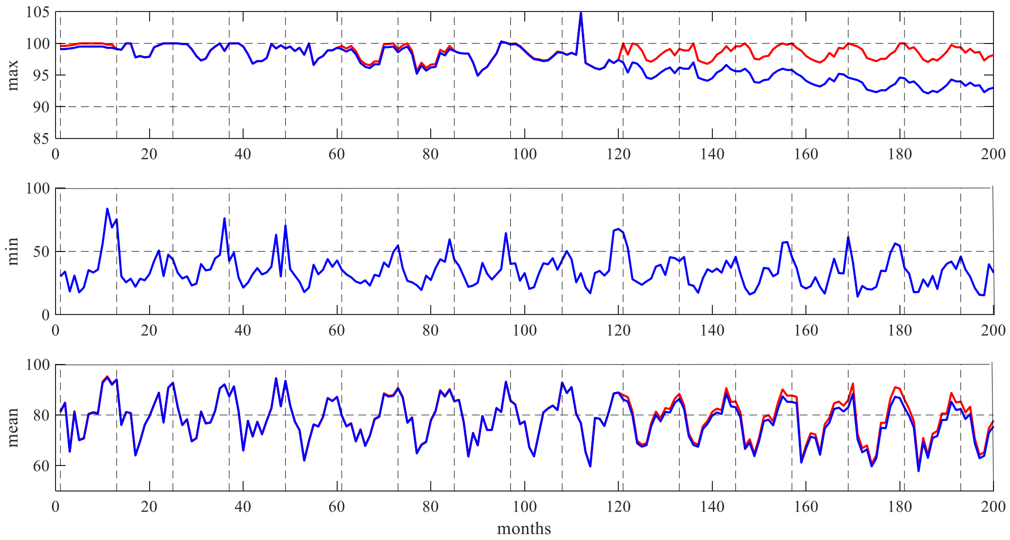

Figure S11: Max, min and mean hourly relative humidity for each month between 2000 and 2016. Blue line is measured value while red line is auto-corrected measurements. In the top subplot, the maximum RH drifts from 2009. This drift is not found on the minimum nor the mean value.

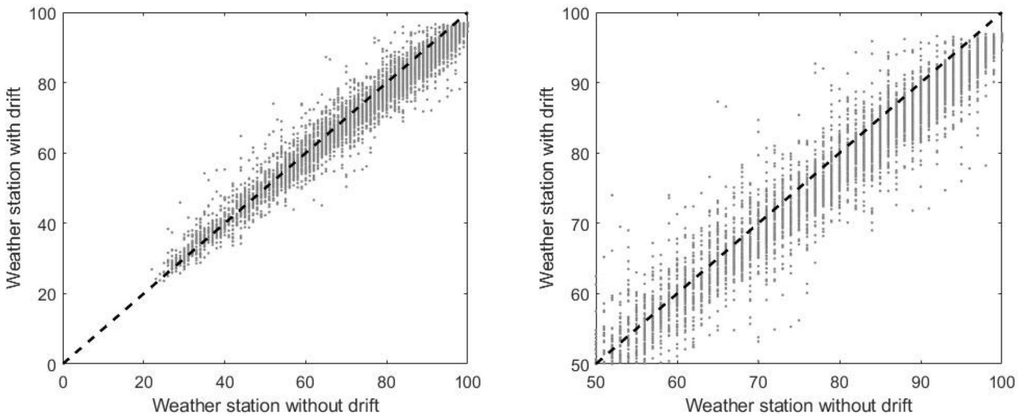

Figure S12: Hourly measured RH values through one year from two sensors placed in the exact same spot, one sensor with a drift and one sensor without.
